# Beyond the traditional distinctions of genome editing: evaluating a vulnerability framework

**DOI:** 10.3389/fgeed.2024.1426228

**Published:** 2024-10-16

**Authors:** Ioanna Angelioudaki, Ana Ruxandra Badea, Martina Bodo, Daniel Fernández-Soto, Emmanouela Sevasti Karyampa, Adam Kokkinakis, Nikolaos Kolisis, Xenia Kominea, Sandra Ozáez Armijos, Simon Vogel, Oliver Feeney

**Affiliations:** ^1^ Second Department of Surgery, Aretaieion Hospital, Medical School of Athens, National and Kapodistrian University of Athens, Athens, Greece; ^2^ Faculty of Philosophy, University of Bucharest, Bucharest, Romania; ^3^ School of Statistical Sciences, La Sapienza, University of Rome, Rome, Italy; ^4^ Department of Immunology and Oncology, Centro Nacional de Biotecnología, Consejo Superior de Investigaciones Científicas, Madrid, Spain; ^5^ Faculty of Science and Engineering, University of Groningen, Groningen, Netherlands; ^6^ Faculty of Forest Sciences and Forest Ecology, University of Göttingen, Göttingen, Germany; ^7^ Department of History and Theory of Law, School of Law, National and Kapodistrian University of Athens, Athens, Greece; ^8^ Medical School of Athens, National and Kapodistrian University of Athens (NKUA), Athens, Greece; ^9^ Department of Fundamental Microbiology, Faculty of Biology and Medicine, University of Lausanne, Lausanne, Switzerland; ^10^ Institute for Medical Genetics and Applied Genomics, University of Tübingen, Tübingen, Germany; ^11^ Research Unit “Ethics of Genome Editing”, Institute of Ethics and History of Medicine, University of Tübingen, Tübingen, Germany

**Keywords:** genome editing, CRISPR, ethics, regulation, vulnerability

## Abstract

Over 40 years ago, the 1982 Splicing Life report outlined the two distinctions that have orientated much of the normative and legal landscape of genetic intervention or genome editing since – that of somatic versus germline (or heritable interventions) and medical versus non-medical (or enhancement) applications. During this time, these distinctions have been used to ethically prioritize some areas of research and potential application, such as somatic treatments, while considering others for prohibition, such as germline enhancements. Nevertheless, somatic interventions may also be done for controversial enhancement purposes while some germline interventions may be done with greater *prima facie* justification (e.g., the enhancement of athletic ability versus the avoidance of Tay-Sachs disease). Even with new somatic treatments that are generally lauded, exemplified with the case of Casgevy, many issues still arise – such as cost and access, particularly salient on a global level. The concerns over a dystopian future of genetic haves and have nots, as a result of enhancement and/or germline interventions, that perhaps may happen, should not distract us from a greater attention to what is happening in the here and now. In this paper, we will highlight the limits of the two distinctions in terms of moving from questions of “should a technology be used” to “how should a technology be used.” We argue that an additional focus on vulnerability and marginalization can be useful to support the attempt to better prioritize which interventions should be permitted or prohibited. We show how this can better dovetail with calls for effective (global) governance and reasonable consensus by focusing on the most urgent issues and developing policy accordingly, while leaving aside more abstract issues for further discussion.

## Introduction

Toward the beginning of the previous decade, advances in CRISPR revolutionized the potential of human genome editing. The revolution was, in short, bound up in the increased ease, efficacy and accuracy of the CRISPR-Cas9 technique compared to previous forms of genome editing and genetic interventions. Various versions of CRISPR and related techniques such as Base and Prime editing, all form the current wave of genome editing technologies that have dramatically increased the success of modifying the human genome. With the substantial increase in scientific and technical possibilities, there has been a related intensity of work on the ethical, legal, and societal implications. Abstract concerns over playing God or interfering with nature as well as concerns over hubris, opening Pandora’s box and irreversibly and negatively affecting what we know as “humanity” have reemerged. However, there are also increasingly nuanced responses emerging alongside the progressively more complex picture of a real, as opposed to hypothetical, biomedical phenomenon. Far from the fringes of He Jiankui, advocacy for heritable genome interventions has grown in recent mainstream proposals for a translational pathway – that can manage risks – toward some limited interventions on the human germline. Issues over inequalities are increasingly grounded not only in concerns over the creation of a genetically enhanced versus unenhanced but on more “mundane” yet intractable concerns – such as limitations of access to emerging and newly approved somatic treatments as well as concerns over the wider costs of responding to this very issue itself (i.e., costs of widening access). Issues relating to the proper scope of treatments versus enhancements versus disability rights continue to be affected both by technological developments and issues relating to “who” draws such lines, as opposed to just questions on “what” and “where” such lines are drawn (e.g., in terms of how involved are persons with disabilities in discussions over what counts as a disease, a disability, or a part of normal human variation). As this technology, and its possible uses, becomes a reality, abstracted ethical discussions are increasingly joined by the societal and political spheres, as forms of public engagement and efforts at achieving coordinated national and international governance intensify. Normative questions are joined with the question of “how” such normative values are to be achieved through regulation “in the real world,” not in the “ideal” society or highly abstracted context behind some arguments. In this paper, we will highlight the limits of the two distinctions in terms of moving from questions of “should a technology be used” to “how should a technology be used.” We argue that an additional focus on vulnerability and marginalization can offer a supplemental framework for prioritizing what interventions are permitted or prohibited, and under what conditions. We show how this can better dovetail with calls for effective (global) governance and reasonable consensus by focusing on the most urgent issues and developing policy accordingly, while leaving aside more abstract issues for further discussion. In the first part of the paper, we explore some of the main themes in the ethical state-of-the-art over the last decade and we start by distinguishing between concerns that are largely ill-defined and arguably hyped, and those more defined and imminent. While, in each case, we assess – like many before – whether the traditional distinctions of somatic/germline and treatment/enhancement capture all relevant normative issues, we also seek to constructively move beyond this with a focus on vulnerability and marginalization. In the second part of the paper, we move toward an area where there has arguably been much more explicit development from the pre-to the post-CRISPR era – that of governance, from calls for public participation and societal consensus to national and international regulatory options. Here too, we can see how a focus on vulnerability and, particularly, marginalization (including epistemic injustice) can be useful frames to assess the *status quo* and what actions are needed to safeguard those most marginalised on national and global levels, and in terms of new developments of misinformation and polarisation. Finally, we make some suggestions for future research and actions.

## An important vulnerability within the two distinctions?

Over 40 years ago, the President’s Commission’s *Splicing life report: social and ethical issues of human genetic engineering* (1982) outlined two distinctions that have orientated much of the normative and legal landscape since–that of somatic versus germline (or heritable) interventions and medical versus non-medical (or enhancement) applications. While these distinctions are not without disagreement and ambiguity, they have been used to ethically prioritize and encourage some areas of research and potential application, such as somatic treatments, while considering others for prohibition, such as germline enhancements. A CRISPR-era example of this can be seen in the hostile reception given to the 2018 case of the early embryonic germline intervention^1^ by He Jiankui (Normile, 2018) versus the positive reaction to the 2019 case of Victoria Gray, who became the first patient successfully treated for sickle cell disease with somatic gene editing using CRISPR-Cas9 (Stein, 2021). These two cases also illustrate how this so-called CRISPR revolution – and the speed of innovation – is adding a greater sense of urgency in focusing such ethical discussions toward forming regulation that is reasonably balanced to permit the emerging benefits of this technology while avoiding some of the ill-considered and potentially harmful applications of the very same technology. Nevertheless, as noted, the aforementioned distinctions are not without disagreement, especially in grey areas or borderline cases (e.g., health-related interventions analogous to vaccines that can be conceived as either treatment or enhancement). Moreover, what are clearly somatic interventions may also be done for controversial enhancement purposes while some germline interventions may be done with greater *prima facie* justification (e.g., the enhancement of athletic ability versus the avoidance of Tay-Sachs disease). The concept of treatments may be no less problematic to persons with disabilities than the concept of enhancements (especially if the former comes with loaded issues of “restoring normality” while targeting a particular section of the population). Moreover, even if one were to stay within the *non-grey* area of *non-controversial* somatic and *unproblematic* treatments, there are many issues that still arise – the sickle cell breakthrough above comes with a hefty price tag (possibly 2 million US dollars per patient^2^), which is particularly salient given the likelihood that the majority of the potential patients for this intervention would be from poorer and disadvantaged sections of the global community. Issues from lack of access, to discrimination for persons with disabilities, to issues facing women and minorities, are all issues that do not seem adequately addressed by those long-standing distinctions. Given the pressing need for ethically grounded guidance for regulation that can supplement the two aforementioned (heavily used but insufficient) distinctions, other frameworks need to be considered in order to further refine ethical-regulatory decision-making beyond permit versus prohibit – but also questions of what, for whom, when and why.

Recalling the idea of “primum non nocere,” before considering how CRISPR technologies can make the world better, it seems at least pertinent to also consider how those same technologies can make the world worse. More precisely, the question is more likely to be one of how we can make the world better for one group of people while not making it sufficiently worse for some that it becomes a problem.^3^ Given the nature (e.g., uncertain long-term impact) of new interventions – whether genomic, economic, societal, environmental, and so on – and given the diversity of sometimes conflicting claims and needs, coupled with (inevitable) limits to resources, it seems morally appropriate to first consider those who are most at risk. It is thinking along these lines that prompts the (World Health Organization, 2021a) to consider it of particular concern to reach out to those traditionally vulnerable and marginalized^4^. Traditionally, vulnerability was generally only used to describe persons in a medical context, e.g., smokers were vulnerable to lung disease. However, vulnerability is a longitudinal, multidimensional, and multilevel process. Experiences of frailty vary based on factors such as gender, age, ethnicity, social class, sexual orientation, and personal characteristics. On the one hand, vulnerability is the susceptibility to involuntary harm due to the human condition, and on the other, it has become ethically understood to be a susceptibility for a person or persons to have their autonomy injured (ten Have, 2015). Vulnerability could be seen as an *inherent* trait of any human being – the susceptibility to get sick and die for instance – and it can also be seen as *situational*, where some groups – for various reasons including natural, chance, and social disadvantages – can be more vulnerable than others (Labude et al., 2022). Moreover, a “person is not “vulnerable” or “not vulnerable:” vulnerability occurs along a spectrum wherein a particular situation or a particular characteristic of a person may place a person at greater or lesser risk of harm” (Gordon, 2020). According to the Declaration of Helsinki, vulnerable groups and individuals “may have an increased likelihood of being wronged or of incurring additional harm” (World Medical Association, 2013). Research participant vulnerability can be in terms of an increased risk of harm and exploitation or due to being a member of a vulnerable group – for instance, if one were a child (World Medical Association, 2013).

Labude et al. (2022) consider the concept of vulnerability – long-standing in bioethics and research ethics (World Medical Association, 2013) – to be particularly relevant to discussions of heritable genome editing. They suggest that greater attention be given to how such considerations of vulnerability are heightened with a focus on heritable genome editing, from additional burdens on women to future children and not just existing children, as well as additional risks of exploitation if coupled with societal pressures to use future interventions, possible marginalization, and stigmatization of persons with disabilities, and unfair access to the benefits of the technology, and so on (Labude et al., 2022). Looking at the broader socio-political dimension, Labude et al. (2022) also note how the notion of vulnerability can also be seen to be linked – especially if without appropriate safeguards – to an increase of societal marginalization (361). For those with increased susceptibility to vulnerability (e.g., health and/or societal) and/or (at risk of being) marginalized, it seems uncontroversial to argue that these persons or groups should then receive special care or special scrutiny with regards to the use of the human genome editing technology (Levine et al., 2004; Walker & Fox, 2018). Labude et al. (2022) have noted how the important role of the concept of vulnerability in bioethics and research ethics should play a greater role in the ethics and guidance of heritable human genome editing. It is not clear why this focus on vulnerability (and marginalization) cannot continue to be broadened and strengthened to better cover a more comprehensive range of ethical, societal, governance and social justice issues regarding all forms of human genome editing – somatic or germline. Importantly, we consider this a focus that may better dovetail with – at least some of – the ethical force that arises from the treatment/enhancement and somatic/germline distinctions. In other words, this ethical force would be strengthened by focusing less on abstract conceptions of humanity and the like, and more on the more concrete effects or risks on those vulnerable and/or marginalized, either directly as a result of the technology itself, or as a result of wider societal impacts arising from other’s use of the technology. This argument is stronger in cases where this focus cuts *across* such distinctions – suggesting some cases where the problem may arise with treatments *as well as (or sometimes even more than)* enhancements, and somatic *as well as* (*or sometimes even more than*) germline interventions. The focus on vulnerability and marginalization can also offer an additional framework for better prioritizing what interventions are permitted or prohibited and under what conditions. By narrowing the “bewildering” field of ethical considerations to those that appear most urgent to address, this should also aid the quest for effective (global) governance and calls for (global) consensus would be most likely to be achieved if the most urgent issues are the focus, leaving aside other areas of disagreement to another day. Of course, even here, issues of vulnerability and exclusion arise in terms of whose voices are heard and whose perspectives are addressed in (global) regulation.

It is not our intention to prioritise “vulnerability” as an overriding value that trumps all others, such as well-established reproductive and scientific freedoms. By offering it as an additional value that can be used to a much greater degree, to supplement (at least) the work of the aforementioned distinctions, we are not making an argument for its ascendency as a sole consideration, nor even a consideration more important to other considerations and values. Depending on the context, sometimes it may be the most important value to be considered, other times not. There may be intractable tensions between this value, for instance, in the context of persons with disabilities and the freedom of others – be they parents or scientists or clinicians. The balance and weighting of various values and priorities will often elude blanket statements (such as often done with the traditional distinctions), but will depend on numerous, and possibly ever changing, variables – such as type and severity of condition, nature of social context of persons involved, other causes of disadvantage, and so on. Tonkens (2021), for instance, considers human genome editing with regards to vulnerable and marginalised groups such as indigenous peoples and how they may be harmed by the overall gene editing enterprise in numerous ways. Sometimes this would require restrictions, other times safeguards or other compensatory measures, or indeed a call for attending to other disadvantages and injustices and not just (or not ever) genome-based ones. Attending to these various vulnerabilities can be done without suggesting that the curtain comes down on human genome editing, although it highlights the possible and emerging harms to vulnerable groups by not doing so. Even if we do not suggest that exacerbating the vulnerability of others is always a/the most compelling reason not to pursue the technology, it seems a minimum mark of respect to highlight the potential and real harms involving such groups. Our task in this paper is not to seek to resolve such tensions, but to add further considerations that give further credence to the importance in using the concept of vulnerability as the first step to start the discussion on addressing such tensions.

Some of this difficulty could also be attributed to the, arguably, vague and ambiguous concept of vulnerability that we use. This could be addressed by focusing on certain vulnerable groups to a greater extent (although we do explore it in relation to groups considered vulnerable, such as persons with disabilities). Nevertheless, this would risk pre-empting a broader exploration of the concept in this new context of human genome editing and the new ways it may create and recreate such categories of vulnerability and of categories of vulnerable persons, and how those pre-existing categories can themselves be understood, recreated and reconsidered in this novel context. Even before a focus on human genome editing, there have been gaps noted in the literature on healthcare disparities related to co-existence of multiple aspects of vulnerability (Grabovschi et al., 2013). A focus on pre-existing categories of vulnerable persons may lose some of the focus on reflecting upon the nature (and gaps in understanding) of vulnerability itself, that contributes to shaping and understanding those categories, and our more exploratory investigation seeks to do this.

The concept of vulnerability is not new, and indeed arose as a special consideration in addition to the four principles rooted in the 1979 Belmont Report (Clark & Preto, 2018). Even if the four principles were considered fundamental, the concept of vulnerability can importantly evaluate a person or groups default or starting position (e.g., a stable advantaged position compared to one marked by precarity) giving greater guidance to additional safeguards to autonomy, avoidance of harm, etc. Some of the more salient instances of vulnerability we explore may be an effect of, for instance, underlying inequalities or past injustice. Nevertheless, such a focus on vulnerability does not simply collapse into a focus on those inequalities or injustice, as the former adds an additional urgency to the safeguarding of the persons affected (past injustice or not). Importantly, what is an effect now can become a future cause, where a vulnerability focus can recognise how the resulting position likely leads on to a continuation of such a situation (e.g., lack of a voice, due to historical injustice, reduces input to the human genome editing enterprise, suggesting the possible emergence of new post-genomic disadvantages and harms (Tonkens, 2021).

## Reassessing the distinctions – what really matters most?

Some of the traditional concerns around genetic engineering – or, more accurately, with the goals of enhancement and/or germline interventions – emphasized the fears of changing human nature, eugenics, and the existential unknowns of the new technologies (Nuffield Council on Bioethics, 2022; National Academies of Sciences Engineeringand Medicine, 2017). Naturalness arguments revolve around allowing natural selection to dictate the development of populations and their traits without interference from outside factors, such as a doctor, religion, or the state (Odzuck, 2018). This concern would be heightened in the context of genome editing, or as some would describe it, a dangerous and hubristic interference in the human gene pool (Habermas, 2003; Sandel, 2007). The notion of wrongful interference would be reinforced when one would view that the target is the human genome, seen by UNESCO (1997) as that which “underlies the fundamental unity of all members of the human family, as well as the recognition of their inherent dignity and diversity. In a symbolic sense, it is the heritage of humanity” (Article 1).

Around the time of this UNESCO Declaration, such strong concerns over genetic interferences were much more, if not entirely, directed toward interventions beyond the limits of therapeutic interventions, as was highlighted by the 2003 “Beyond Therapy” report, which notes when:

The goal is restoring health, the doctor’s discretion is guided by an agreed-upon and recognizable target. But a physician prescribing for goals beyond therapy is in uncharted waters. Although fully armed with the means, he has no special expertise regarding the end—neither what it is nor whether it is desirable. To the extent that the patient is transformed from a sick person needing healing into a consumer of technical services, medicine will be transformed from a profession into a trade and the doctor-patient relationship into a species of contract, ungoverned by any deep ethical norms (Kass, 2003, 304).

It is particularly evident in this contrast between agreed-upon and recognizable health goals and a largely ethically ungoverned form of consumerism that such concerns over unnatural interference do not, in general, arise from a focus on treatments, but that of non-medical or enhancement applications. However, at least some of the “unnatural interference” arguments such as concerns over enhancements, are not necessarily strongly correlated to the current genome editing technologies’ ability – whether now or perhaps even in the future. The idea of “designer babies” is criticized as unhelpfully inaccurate, since it is a term that fails to include the difficulty of predicting and controlling a set of human characteristics, such as intelligence, that are influenced by multiple genetic and environmental factors. Moreover, although the alarming idea of designing human characteristics based on the idea of “perfection” is rooted in a history of eugenics and enhancement, it can’t yet be easily applied to the technology of human genome editing. Most of the human characteristics that could be the target of enhancement methods are influenced by a large number of genes that would be unsafe or perhaps even effectively impossible to control using the current CRISPR technology and other environmental factors (Daley et al., 2019). In the end, there will be some speculated interventions that will remain science fiction. For interventions that are potentially genuine matters of concern, it is wise to ethically anticipate what future problems may arise, without prohibiting all other areas of interventions, especially if some of the interest in prohibition derives from the more abstract, removed and possibly even science fiction concerns. This is not to say there are no issues arising in the context of sports and gene doping (World Health Organization, 2021b) – but these seem to be familiar problems with a less than fundamental restructuring of the concept of humanity at stake. Some extreme enhancements, that raise concerns about perfection, may not be likely, especially in the medium term. However, a focus on such potential future enhancements may act, unwittingly, as a distraction from actual enhancements that creep in, via medicalization, into the language of treatments. They could be modest forms of enhancements – perhaps to slow age decline, or to raise cognitive levels at borderline treatment/enhancement cases – or might be similar to preventative interventions. Society may never approve “enhancements” as such, but perhaps some will, gradually, in the guise of treatments via medicalization. Even if we can identify medicalization as it happens, it is another step to distinguish well-founded forms of medicalization from over-medicalization (Kaczmarek, 2019).^5^ Medical progress may push the boundaries on what was accepted as normal and natural at one time, but which at another time may be considered preventable health problems (for instance associated with prolonging quality of life into old age). Even when one deems certain forms of medicalization to be negative, the idea of isolating enhancements and not treatments or preventative interventions, is problematic in particular at the level of medicalization – as it will be difficult to prohibit some enhancements without prohibiting some treatments (Juengst et al., 2018). This is not to say we should not use the word “enhancements,” but that is not clear that such a treatment-enhancement distinction – and the extreme moral weighting it represents – will be fruitful in many areas in the nuanced context where genome editing interventions are actually emerging. At least on its own, it seems less useful than at the level of broad blanket judgments. The potential of the category of “prevention” to undercut the conceptual separation between treatments and enhancements is compounded by how it can undercut the separation for the purposes of governance (Waltz et al., 2024). The grey area of prevention can give rise to “incidental enhancements” – whether by off-label use or, more centrally, in later-life interventions where prevention – by blurring effective distinctions between cellular senescence and normal life spans (Waltz et al., 2024). However, for instance, if we viewed some age-related enhancements with improved quality of life versus some highly competitive enhancements that would increase inequalities, a vulnerability focus would seem to encourage the former (better life for an otherwise vulnerable group) while preventing the latter (where inequalities would adversely affect the most vulnerable in society). In such cases, some enhancements would not be problematic while others would, thereby undercutting the use of the treatment-enhancements distinction entirely in such cases – while highlighting another focus (on vulnerability) that may have primarily been at work.

Just as *some* enhancements may not be problematic, *qua enhancements*, some treatments might not be *un*problematic just because they are treatments. One of the often highlighted skepticism by the disability community towards the potential use of gene editing is the so-called expressivist argument; the view that morally or legally approving genetic intervention for reasons of “cure” (in other words for “health” and not for “enhancement” purposes) will create the impression – express the attitude – of an adoption of eugenic policies or attitudes discriminating against certain people with genetic traits deemed to be disabling in the current society’s environment (for example, see Boardman and Thomas, 2023). It follows, as this argument suggests (or more specifically, as critics of this position usually present it to suggest) that a state policy allowing interventions based on a medical definition of “normality” will consider disabled individuals as “abnormal” or “defective” or leading lives “not worth living.” These are terms with a very heavy historical meaning – urging, thus, prospective parents to choose genetic interventions in case a certain genetic condition defined as “curable” is detected, say, by IVF-PGD or other forms of screening. This choice, when or if rendered common, will simultaneously create a social environment in which prospective parents who do not act accordingly might be considered reckless, irresponsible or (using terminology from the political philosophy of egalitarianism) bearers of “expensive tastes;” as their choice not to proceed to a genetic intervention because of their conception of life “worth living” for their offspring does not coincide with the widespread social practice and therefore, society should not bear the economic burden of creating a hospitable environment for “disabled” individuals. This is not to say that this negative scenario will be the outcome, but that it is a concern for many who are considered disabled (Parens and Johnston, 2019). It might very well also be exacerbated by widespread enhancements ratcheting up the general average of “normal functioning,” and further increasing issues of accessibility for those currently facing forms of disablement while possibly adding more from the current (so-called) normal range of abilities (Buchanan et al., 2000). However, in such cases, speculation – even if well grounded – is not a certainty. In terms of the expressivist objection itself, it is not felt by all persons with disabilities to be an overriding factor (for instance to restrict freedom of others to avail of such technologies for their children) – indeed, its very nature is much more complex than initially appears (Boardman and Thomas, 2023). Sociological research will be needed to monitor these post-genomic social changes that may happen due to the introduction of this new technology, with a focus on how it affects vulnerabilities. Beyond simple, and sometimes simplistic, blanket recommendations of permitting unfettered freedom or prohibition, the focus on vulnerability can highlight various measures and appropriate safeguards, particularly by bringing the voice of persons with disabilities more centrally to discussions of the development and governance of genome editing technology – whether for treatments or enhancements (Parens and Johnston, 2019).^6^

Nevertheless, it is worth noting again that it is not clear if the concept of “enhancement” is the cause of more concern than the concept of “treatment.” For one thing, it is comparing the hypothetical with the actual, respectively. For another thing, from the perspective of a person with disabilities, treatment is a key concern, not (just) enhancements. In fact, an enhancement technology aimed at improving *everyone* in society may be a more preferable focus than a therapeutic technology in terms of the messages they may elicit. It is treatment, not enhancement (or much more than enhancement) that aims to define or identify a subset of persons to be considered as sub-normal in some relevant respects, with the aim of moving such persons to the level of “normal.” The expressivist argument would take hold in the latter scenario, while less so in the former.^7^ So in one (arguably hugely important) respect, the concept of enhancement is *less* problematic than the concept of treatment. While persons with disability may not consider themselves intrinsically medically vulnerable – at least not to the level forwarded by the medical model of disability – this model would still admit the broader relevance of the notion of vulnerability, not simply due to medical causes, but due to their interaction with disabling sociopolitical factors (Parens & Asch, 2000). This concept, particularly combined with the marginalization of the disabled voice from many discussions, seems a necessary additional principle beyond simply questions of treatment versus enhancement.

In terms of the somatic versus germline distinction, there are many reasons to be wary of the latter – at least when we are speaking of germline as heritable interventions, and not the less problematic basic research. From the possibility of permanent off-target mutations and mosaicism and other unforeseen consequences for future generations – who cannot consent – the level of risk is such that there are currently few compelling reasons to rush into making heritable changes, such as done by He Jiankui (Ranisch et al., 2023). Nevertheless, there may be such reasons in the future, and as the basic research continues, the risk-benefit weighting may be such that it is a viable option in some cases (Ranisch et al., 2023)^.^ In this paper, we do not seek to comment on all such concerns nor make any such strong claim that concerns over germline interventions are overblown compared to somatic interventions, in every case. However, as with the therapy-enhancement distinction, we further question the robustness of the default weighting against germline and toward somatic (beyond fuzzy boundary issues), in every case, and without an additional consideration, such as a stronger focus on vulnerability.

One area where this can be seen to be evident is in the context of the fear of post-genomic inequalities arising from differential access to heritable genomic technology (Feeney, 2012). An extreme example of this concern could be seen in the pre-CRISPR era work of Maxwell (Mehlman, 2003; Mehlman, 2005). Heritable enhancements, he argued, could give rise to a “genobility” that would destroy the foundations of democracy, and which required a strong prohibitionist response, including ill-defined monitoring of people’s DNA, and the use of military force and sterilization of “persons whose germ line has been enhanced so they cannot pass on the modifications to their children” (Mehlman, 2003, see also Feeney, 2010). It is unlikely that such suggestions would be taken seriously by many who would otherwise be concerned over post-genomic inequalities and who would take a more moderate prohibitionist stance as a consequence of this concern. Such discussions may have greater traction in a time where the underlying technology of genetic or genomic modification was considered to be many years in the future, but they are less useful now – especially in terms of helping to form both moral and legal guidance for more modest possibilities that may arise in the post-CRISPR era. What this also highlights is how one should be careful not to be distracted entirely by the more abstract, even if still genuine, future possibilities (e.g., genobility) while not adequately addressing current issues of inequality. For instance, inequitable participation in research is demonstrated by the underrepresentation of minority patients posing not only a problem for justice but also hindering the understanding of the correlations between genetic variants and diseases in specific populations, placing limitations on the progress of science. In this way, inequality is perpetuated, as it subsequently provides different access to research (Doxzen & Halpern, 2020). In some cases, patient underrepresentation is rooted in historical events. Minority groups in the United States, especially African Americans, have faced generations of mistreatment that may perpetuate mistrust of both biomedical research and science as evidenced by their low rates of enrollment in research (Corbie-Smith et al., 1999; Gee & Ford, 2011; Institute of Medicine et al., 1995). Therefore, the reduced participation of minorities in research actions is likely to lead to their underrepresentation and possible benefits that will be obtained through them. More specifically, without a comprehensive understanding of the genetic background of these individuals, it will be more difficult to adapt a potential treatment (Doudna and Charpentier, 2014). When a treatment receives the appropriate approvals and enters the market, access to it is likely to be prohibitive for people belonging to minorities. The problem is exacerbated when one combines overt or subconscious racism and differential treatment in medical care (Hildebrandt and Marron, 2018).

Concerns over a future genobility via germline enhancements, should not distract us from the more concrete and immediate problem of justice that is situated in the context of somatic treatments. The success story of Casgevy – the world’s first CRISPR-Cas9 gene editing therapy – has been granted regulatory approval for the treatment (one could tentatively say “cure”) of sickle cell disease (& β-thalassemia).^8^ However, this success is, or should be, heavily qualified by the issues of justice and access that it highlights. With a price tag in the millions of Euros or US dollars, the cost of this therapy is significant, especially given the disadvantageous economic position of a large portion of the global sickle cell disease sufferers. Any arguments for expanding access to this therapy would have to question how much this would cost vis-à-vis other healthcare priorities, particularly for the worst-off and most vulnerable in society – at a national and global level. For reasons of justice, one may reasonably be more concerned over interventions that are somatic and that affects so many, as opposed to issues of justice in the context of heritable interventions (or enhancement for that matter) that may only ever affect relatively few. At this point, one could highlight a general utilitarian point in terms of justice, but also in terms of ethical considerations more widely – numbers count. There are – and will likely always be – far more people affected by somatic interventions, rather than by heritable interventions.^9^ Of course, not only numbers count, but the severity of the condition or disadvantage behind those numbers count.^10^ In terms of justice in access, this special focus on the vulnerable or marginalized overlaps with an aspect of Rawlsian political theory, where a just society is one not necessarily with greater equality, but one in which inequalities are only permitted if to the greatest absolute benefit of the worst-off situation (Rawls, 1971/revised edition 1999). In the context of emerging developments in genomics, Colin Farrelly (2016) has advanced a broadly prioritarian approach which similarly focuses upon the worst off but to a less stringent degree (for instance, taking account of non-ideal factors such as the costs of access for all coming at the cost of other healthcare priorities). In such cases, the worst off, or least advantaged, need to be identified in some way to command a reasonable consensus over who they are and here again the notions of vulnerability – not just the aforementioned distinctions – seem a key focus to this end. Moreover, if significant numbers of the worse off, and most vulnerable, could be better served with a wider roll out of somatic interventions, by permitting a private pursuit of enhancement or germline technology to the better off (with a proviso that through funding and innovation, this funded the wider somatic access for the worst off), one would need to make an argument stronger than speculating that a genobility may arise in the future. Of course, this is not to suggest that there are no such arguments to be made.^11^ However, whether or not they are treatments, enhancements, somatic or germline, does not seem sufficient to do anything other than beg the question.

## Public engagement – vulnerability and marginalization

It seems less “question begging” to focus on how such interventions, and the associated research, affect the most vulnerable. As noted above, protection of the vulnerable is a key consideration in international ethical principles regulating human research such as the Declaration of Helsinki (World Medical Association, 2013 - DoH sections 19 and 20). Of course, one could also see the broader form of vulnerability intrinsic to the research-research participant relationship being addressed by the general principles of the Declaration. However, recalling the aforementioned Splicing Life Report, a good example where a greater focus on vulnerability may have been needed, could be seen in the “Cline controversy.” Martin J. Cline was a US clinical researcher who attempted unauthorized gene therapy treatment of two thalassemia patients with genetically altered bone marrow cells (President’s Commission for the Study of Ethical Problems in Medicine and Biomedical and Behavioral Research, 1982). The experiment attracted significant criticism from the scientific community, resulting in, among other things, the loss of substantial funding. Notably, the main ethical issue revolved around the right timing to initiate trials of human gene therapy after animal work had been carried out. There is no mention about the risk that Cline’s actions exploited the vulnerability of the individuals with beta-thalassemia who may have been desperate for a cure.^12^ In fact, the word “vulnerability” is completely absent from the Splicing Life Report in its entirety and only appeared in revised versions of the Declaration of Helsinki from the turn of the century (President’s Commission for the Study of Ethical Problems in Medicine and Biomedical and Behavioral Research, 1982; World Medical Association, 2000). Labude et al.’s focus on a greater role for the concept of vulnerability, as applied to He Jiankui’s germline experiments, can be seen to apply equally to Cline’s somatic human trials too.

It is also noteworthy how at the time of the Splicing Life Report, public engagement in scientific decision-making was not as formalized or emphasized as it is today. The Report’s ethical debate mostly focused on the roles that scientists, regulators and policymakers played in ensuring safety, minimizing risks, and promoting public welfare. This is not to say that the broader public was completely ignored; however, the focus revolved around its education with the aim to increase their understanding of the capabilities and the potential of the technique, lacking specific recommendations or strategies for genuine public engagement, especially of those most vulnerable. The contemporary focus would more likely include such perspectives (although not guaranteed). In this respect, human genome editing could be seen not only as a scientific endeavour, but one that is guided by the community in which it will be used, given legitimacy not only by those who administer and regulate its use, but also, and maybe more importantly, by its final consumers (Mohr and Koch, 2016). Therefore, both somatic and germline HGE require “opening up a space of possibility in which relations between the state, its institutions, and its citizens can be negotiated” (ibid). This shift reflects a broader understanding of science as a social endeavor and the recognition that public values and perspectives should be considered when shaping the direction of scientific research and innovation.

Even though there is no unequivocal definition for public engagement since the term is widely used in different sectors, based on the National Co-ordinating Centre for Public Engagement (NCCPE) *“engagement is a two-way process, involving interaction and listening, with the goal of generating mutual benefit.”* But what is the level of engagement social scientists and ethicists should aim at? And will there ever be “enough” public engagement? The human genome is considered the common heritage of humanity’, a global public good^13^. It is therefore impossible to believe that advancements regarding HGE can be made without consulting the general global public. And whilst science is a starting point, it could be argued that it is up to the people to ultimately decide which values and worldviews ought to be protected and which can be sacrificed. Even bioethicists fall short when it comes to deciding what is ethically right or wrong in a global context of pluralism. To some degree, a focus on vulnerability and the effects on those worst off may have the potential to generate some form of reasonable consensus, while leaving many areas of disagreement for another day.

Despite uncertainty over its future role of human genome editing, its social implications are already so far-reaching and complex that managing them requires more than just technical expertise (Scheufele et al., 2021). The scientific community has a long tradition of self-regulating, engaging with social scientists and the lay public retroactively, once the technology is in its last stages of development. But this mechanism has been largely questioned, especially following Dr. Jiankui’s announcement of CRISPR-edited babies being born. Soon thereafter, prominent scientists all over the world called for an international moratorium on editing inherited genes. Whilst the core idea has been to create a more just and responsive governance through public engagement, there are still significant gaps between the conceptual and the empirical framework (Conley et al., 2023).^14^ The debate over the global governance of genome editing is currently dominated by five organizations: ARRIGE, National Academies (US and UK), WHO, EGE and the Global Citizens’ Assembly on Genome Editing (GCA). Out of these five organizations, only the GCA can be considered a citizen deliberation-driven, bottom-up model featuring two-way interactions with community-based organizations in the area. The rest, despite their mission statements, fail to move on from expert-driven, top-down models with the debate being shaped predominantly by scientists, policy experts and civil society groups.

If it is true engaging the public comes with a new set of challenges, including resolving conflicts and contradictions that inevitably arise when pursuing deliberative democracy in the context of science and technology; closing the door to public engagement is not necessarily better as we risk falling into the realms of epistemic injustice and selective ignorance. Epistemic injustice occurs when someone’s capacity to know things and to be perceived by others to know things is harmed, and it can manifest in two primary forms: testimonial injustice and hermeneutic injustice (Fricker, 2007). On the one hand, testimonial injustice occurs when a hearer treats someone with greater skepticism than they normally would due to biases (misogyny, ableism, etc.) regarding that person’s identity. In the context of gene-editing, vulnerable groups face testimonial injustice when their concerns and experiences are dismissed or undervalued. And this can occur at the doctor’s office as well as in public forums, leading to a systematic exclusion from important decision-making processes. Moreover, not being perceived as credible sources of information makes it harder for patients to further their cause: not only are they undermined by others, but they also lose confidence in their own ability. It would be no surprise if they gave in to therapies proposed by doctors just because they believe it’s their only option. On the other hand, hermeneutic injustice occurs when there is a gap in our shared cultural resources that puts someone at an unfair disadvantage when making sense of their lives. In the context of gene-editing, this could show up as some marginalised groups may not have access to the language, concepts or frameworks necessary to properly express their worries or the effects they believe HGE could have on their lives. This gap can lead, once again, to their exclusion from meaningful participation in public engagement processes, further entrenching their vulnerability. The failure to address these forms of epistemic injustice in discussions around HGE risks perpetuating selective ignorance, where only certain perspectives are deemed valid while others are systematically ignored. This is particularly problematic when the goal is to pursue deliberative democracy, as the intention is to incorporate a wide variety of viewpoints and opinions in the process of decision-making. Therefore, if epistemic injustice is not addressed, the public engagement process may actually strengthen existing power imbalances, rather than challenging them.

The Nuffield Council on Bioethics (2022) has acknowledged issues regarding marginalization of disabled individuals based on the recent advancement in prenatal screening technologies. However, both the Report by the National Academy of Sciences and the National Academy of Medicine, and a Position Paper by the WHO fail to highlight the importance of vulnerability regarding the research on heritable HGE. Recalling Labude et al. (2022), some vulnerable groups to be considered are the ones that could be directly affected by human genome editing, such as prospective parents, as well as the women that will be potentially involved in the process of IVF, and their prospective children. Acknowledging the concerns of these underrepresented groups is crucial for creating a safe environment in which public engagement together with research can flourish (Labude et al., 2022). Mónica I. Feliú-Mójer (2020) highlights the importance of cultural understandings as the basis of effective engagement, so we can consider the perspectives and values of each underrepresented community, as opposed to the current predominance of the Western world. Feliú-Mójer points to the absence of a strong sub-Saharan African scientific involvement in malaria CRISPR research, that results in the underappreciation or even absence of the point of view of the community that is actually affected by the disease. The above is only an example of the possibility that this cutting-edge technology could be developed and used by only the privileged, leading to epistemic injustice and ignoring vulnerable groups that could potentially benefit from it.

## Governing with ethical disagreement/ethical polarization

International governance of HGE technologies in the form of a comprehensive, global and legally binding mechanism has surfaced lately as a crucial matter of discussion and concern, including in the World Health Organization, 2021a *HGE: A Framework for Governance* (World Health Organization, 2021b) and in its *Recommendations* (World Health Organization, 2021c). Given the various challenges HGE brings to humans and societies (e.g., as individuals and as members of dynamic societies/environments), scientists and stakeholders are engaged in addressing the issue from a governing and regulatory perspective. We suggest that, despite the many ethical/scientific challenges and political polarization, several practical steps regarding HGE technologies can be taken, such as enhanced domestic dialogue, international cooperation and verification talks, which could be more meaningful for (and better caters to the needs of) vulnerable groups than an overarching international treaty or convention.

At present, the governance of human genome editing (HGE) is a combination of international ethical guidelines, domestic laws and regulations, a partially implemented legally binding convention^15^, and an attempt at international collaboration under the WHO and other similar fora. While vulnerable and marginalized groups are not specifically addressed, the overarching goal of these mechanisms is to prevent an environment or conditions that might foster vulnerability, whether this means protection from abuse or fair access to treatment. For instance, in the latest “Framework of Governance” report by WHO, there are concerns stated about clinics that turn therapy into unscrupulous entrepreneurship, advertising and promoting unproven genome editing therapies that target and eventually threaten vulnerable and marginalized populations. Such ventures would definitely put at risk any legitimate scientific effort for “ground-breaking treatments.”

Currently, the protection of certain vulnerable groups is ensured through a separate set of international instruments, among which are the (United Nations, 2006; United Nations, 1989), or through national guidelines and legislation, as is the case of the U.S. National Institute of Health (NIH) Office of Research on Women’s Health (ORWH). While these instruments, when adhered to, have provided sufficient protection to vulnerable groups, it is possible that they might not satisfy the needs of post-CRISPR society, and allow oversights as was the case with the Chinese twins when Dr. He Jiankui exploited the double vulnerability of pregnancy and HIV. The first reaction to a greatly unethical and unregulated HGE intervention was to China’s gene-editing scandal, and that was the reason why its criminal code was updated to include a ban on changing the human genome. However, to avoid such strict and downright prohibition of any HGE, a ground for compliance should be set to include a reporting mechanism for unethical studies (Schaefer et al., 2021). Nevertheless, it should be based on regulations that avoid stigma and promote the values of accountability and scientific integrity. Given the serious and widespread implications that HGE can have for societies, human health at large, and even national and international security (Townsend, 2020), moving towards a global, harmonizing and legally binding regulating framework on HGE could be the safest way forward (World Health Organization, 2021a), but until such a global, legally binding mechanism is reached, there is still work that can be done to consolidate HGE governance.

Ideally, such an endeavor would mean moving beyond political interests, power differentials and ethical disagreements to establish common ground and principles onto which the international community can build such a framework, engage in its conduct and achieve results. Moving in that direction, discussions are open for reevaluation and redefinition of the concepts of the fundamental ethical principles of respect for autonomy, beneficence, non-maleficence, and justice, adjusting to the novel demands. For instance, challenges coming from increasing healthcare costs and growing economization of medicine, in combination with rapid knowledge advancements on HGE in the context of international collaborations are transformative and require updates on the above often competing values and priorities in such biomedical applications as well as specifications on the ways they can materialize. At the same time, input from the complex cultural values and social belief systems/views (e.g., pluralist publics with differing religious sensibilities and worldviews) towards the emerging HGE-oriented applications should be regarded in structuring the governance networks (Nelson et al., 2021). Besides, concepts of persons, rights, responsibilities and cultural or other identities implicate how individuals function in their society and put their decisions into action.

Given the deep polarization and increasing political divide between states and poles of power, the need for dialogue and transparency seems to be greater than ever in the post-Cold War era. The potential of human genome editing to drastically alter medicine, human life and international security makes it a viable yet benign topic for such diplomatic conversation. As the World Health Organization emphasizes in its 2021 technical document “Human genome editing: recommendations,” one of the near-future priorities should be to enhance the HGE dialogue at both domestic and international levels (World Health Organization, 2021b). At the domestic level, an adequate conversation on HGE activities should include all relevant agencies and stakeholders, such as government officials, health departments, state-owned and private laboratories, academics, ethicists, patients, prospective parents, vulnerable groups, and NGOs. The end goal of such a conversation should be to prepare to deal with the current and potential uses of HGE technologies, by ensuring equitable access and treatment for all groups of society. Only such an inclusive approach at the national level would make a comprehensive international collaboration possible (Townsend, 2020).

International/transnational cooperation can have benefits beyond sharing information between scientific communities and regulatory authorities. For example, it can encourage coordination of regulatory standards and procedures or can promote respect for divergent national policies and cultural contexts (National Academies of Sciences, Engineering, and Medicine, 2017). In other words, it is possible that international cooperation might support vulnerable groups and prevent conditions leading to an HGE-induced vulnerability by actively exchanging information and sharing examples of best practices. International engagement on HGE bioethics is carried out through fora such as the *International Summits on Human Genome Editing* and the National Ethics Committees (NECs) Database, a WHO portal reuniting national/regional offices and an array of ethical standpoints. Out of 124 NECs in 100 countries, 44% are in European and 47% in high-income countries (Hummel et al., 2021). However, there might be added value in approaching the topic of HGE at a higher diplomatic level and encouraging states to be transparent about their scientific and political positioning on the issue.

Irrespective of the effectiveness of national legislation or global regulation, effective governance should eventually include global oversight or a means of verification and compliance. Over the years, similar technologies, such as atomic energy and the manipulation of biological agents, have demonstrated that successful regulation requires a working verification mechanism. Therefore, regardless of whether HGE activities will be regulated through existing frameworks or completely new ones, an international verification system should be the ultimate goal, since regulation alone does not suffice. Objectives of verification typically include transparency (e.g.,: information exchange/sharing), confidence building (e.g.,: peer-reviewed missions of HGE facilities), and support for research and development (R & D) through expertise, lessons learnt, best practices etc. These would also support initiatives for the dissemination of knowledge and discussion of concerns among multiple levels of governance: national/federal, international. The pending question is whether a HGE verification mechanism would be achievable in today’s polarized world. And if it would indeed be feasible, what role would it play within the vulnerability framework? Even though there is no easy or definite answer, precedent tells us that a global legally or ethically binding verification mechanism is more likely to offer safeguards against HGE misuse or abuse in vulnerable groups or against a structural context of vulnerability than solely a treaty/convention which would have to meet the lowest common denominator (or minimum harmonization in EU legislation). As in most other high-stake fields (chemical, nuclear, missiles), international verification of HGE activities would rely on the “mutual benefit” principle and might be considered a win-win strategy if the stakes are high enough. This would not necessarily start with a legally binding mechanism, but a “soft law” instrument such as a code of conduct (Marchant, 2021), where verification would initially be voluntary. In this regard, a practical starting point for a potential HGE code of conduct and, ultimately, an oversight instrument could be the *compulsory* registration of all HGE preclinical and clinical trials in the HGE registry set up by the WHO (World Health Organization, 2024), which already aims to be a transparency mechanism. At the moment, registration is voluntary, although WHO states that “failure to register any work that falls within the scope of the HGE Registry must be considered as a fundamental violation of the principle of responsible stewardship of science” (World Health Organization, 2024).

## Setting a revised framework for the future

Most of the ethical discussion about genome editing has pivoted around two distinctions: somatic versus germline interventions and treatment versus enhancing applications. Although still useful, the current context requires moving beyond what has become an oversimplification: somatic interventions have been linked to medical purposes and have therefore been encouraged, while germline editing has been halted because of the risk of enhancement. Both here, and over a now extensive literature, have been shown to have certain crucial limitations – but the key issue is how to proceed from that understanding. The somatic-germline distinction must be informed by the limitations of the technique. Most data point to the important possibility that babies born through genome editing could be genetic mosaics (Raposo, 2019), and/or heterozygous for the desired mutation (Greely, 2019). These limitations imply that some so-called germline interventions could potentially miss the germline cells and thus be non-heritable, similar to somatic interventions, or at least only partially heritable. The distinction between a medical intervention and an enhancement has never been an easy one. A prevention-oriented intervention can be questioned, as has been the case for Dr Jiankui’s modified babies: the genetic change aimed at conferring HIV protection for life and not only from infection at conception, which is normally minimized in IVF routine (Greely, 2019), and it has been later linked to enhanced cognitive function after stroke (Joy et al., 2019). As highlighted by this particular case, the unknown effects and uncertainty makes it difficult to make a definite claim on the enhancement nature of a given modification. In addition, although sometimes overlooked, somatic interventions can have enhancing potential, as it could be the case of enhancement for sports competitions. In fact, the World Anti-Doping Agency, 2024 has included “gene and cell doping” as prohibited methods at all times (in- and out-of-competition), as many targets have already been identified with this potential (Barton et al., 2002; Wang et al., 2004).

Thus, we argue that new principles are needed to complement the existing ones in the human genome editing discussion. Here, we propose that a vulnerability framework could be useful in the current state of the conversation. A focus on vulnerability could be achieved at different levels:

### Vulnerability in the subject of the intervention

Genetic diseases are usually rare and people suffering from them might be vulnerable and prone to take further risks with less information. The informed consent, key in any medical interventions, has some particular difficulties in the case of genome editing. For instance, in germline editing, the genome being modified belongs to an individual who does not exist yet, and as such cannot give consent. In the case of somatic editing, off-target effects might be difficult to include in an informed consent form due to their unpredictable nature but should be fully communicated and understood anyway by the patient for proper consent. All things considered informed consent forms would benefit from incorporating a vulnerability framework (Strickler and Havercamp, 2023).

### Vulnerability in parents (for germline editing)

In germline genome editing, the parents are the main subject of the intervention, which has reproductive purposes. A couple wanting to have children but fearing they could inherit their genetic disease is itself in a vulnerable position and might be prone to suffer epistemic injustice. Apart from the need for proper informed consent, as addressed before, consideration should be given to the possibility that parents might be vulnerable in terms of their freedom to choose whether or not to perform an intervention on their offspring. If genome editing might become widely available, vulnerable parents might be pressed to opt in, as the contrary could be perceived as an irresponsible decision (Nov-Klaiman et al., 2019). This applies to somatic interventions in children as well, as in the case of vaccination. On the other hand, if genome editing becomes available but remains a heated public debate, parents might be vulnerable to misinformation and to the so-called informed ignorance (Cohen and Garasic, 2024) and could decide to opt out on the basis of hyped concerns, as discussed before.

### Vulnerability in communities

Genome editing has the peculiarity that it tries to avoid genetic conditions, which might be perceived as part of an individual or a community’s identity. In addition, what is normal in a population is ambiguous and subject to change over time and under different circumstances. Whether one specific deviation from normality constitutes disease is not straightforward and should be evaluated carefully. Many individuals in the deaf community, for instance, reject the notion that they need to be “cured,” as was shown when cochlear implants became available, going as far as to say that cochlear implants represented a form of attack against a minority culture (Sparrow, 2005). On the contrary, some have argued that if deafness became correctable, individuals/parents refusing to correct it “would lack the moral right to demand that others pay for costly accommodations to compensate” for it (Tucker, 1998). It should also be noted that people suffering genetic diseases, as minorities, could be vulnerable in terms of being denied their treatments and condemned to suffering by the moral standards of a majority. For these reasons, any genome intervention, whether germline or somatic, should be evaluated in terms of its effect on vulnerable or potentially vulnerable communities (Parens & Johnston, 2019; Tonkens, 2021).

### Vulnerability in the design of the target

The molecular target of genetic interventions is usually designed using available reference genomes. Considering the genetic diversity of the human population, the inclusion (or lack of) of vulnerable communities in the reference genome might compromise the universality of the treatment, in terms of safety or efficacy. For instance, one study found that the newly approved treatment Casgevy has a potential off target produced by an allele common in African ancestry, overlooked by previous analyses (Cancellieri et al., 2023). This issue, discussed during its evaluation by regulatory agencies (European Medicines Agency, 2023), is especially relevant as the disease targeted (Sickle cell disease) is particularly common among those with African ancestors (Centers for Disease Control and Prevention, 2024). This example shows the importance of considering vulnerable populations, and their genetic diversity, when designing genetic interventions.

### Vulnerability on a global scale

The discussion on genome editing must be truly global, accepting that there is no cultural uniformity and avoiding the imposition of Western ethics. It should be considered how global decisions on the matter may affect vulnerable countries. For instance, a ban on genome editing in developed countries could make developing countries with no legislation on the matter vulnerable to circumvention tourism, a type of health tourism (Rosemann et al., 2019; Cohen, 2014). On the contrary, if genome editing was to be approved globally, affordability problems might be inevitable and they could deepen the development divide between countries, translating economic differences into health differences. As noted, unequal access to this technology is already a global problem for the approved somatic genome editing treatments. We suggest that an international verification mechanism, such as the one set for instance for nuclear power, might be an appropriate way forward. Additionally, when discussing the benefits and risks of genome editing, a focus on vulnerability would require taking into account the difference in the quality of life of different genetic diseases across countries (Kats et al., 2024). Otherwise, there is a risk of underestimating the benefits of the technique in vulnerable settings in developing countries, as certain genetic diseases might seem more bearable to those in a developed country living under privileged conditions.

It should be noted that there can be tension between the different categories of vulnerability, for instance the interest of vulnerable parents might be different or even opposite to the interest of vulnerable disabled communities. Furthermore, tension can also arise between vulnerability concerns and other ethical considerations, for instance protecting minorities from stigmatization due to genetic interventions might conflict with the right to enjoy the benefits of scientific progress (recognized internationally in the International Covenant on Economic, Social, and Cultural Rights). In some cases, harm – individual or societal – may be sufficiently severe that prohibition is justified but, other times, the benefit forgone to others would be too great. Tensions between ethical principles are not uncommon in clinical practice (Varkey, 2021), and their resolution is not straightforward, requiring the incorporation of new perspectives into the conversation. Importantly, we do not propose the vulnerability framework as a stand-alone factor, nor argue that it should be prioritized over other principles. Rather, we postulate this framework as a complementary approach to improve the ethical discussion and to ensure that vulnerability concerns are heard and pondered together with other ethical concerns.

## Conclusion

It is not a novel step to criticize the two long-standing distinctions that have orientated moral discussions from the days of recombinant DNA technology to CRISPR gene editing. Nor is the purpose to dismiss their ongoing usefulness and value. Generally, we hope to further highlight the reduction in applicability or relevance of the treatment-enhancement and somatic-germline distinctions in the increasingly complex context of fine-tuned questions on “how” to use the technology and its various applications, in various situations, beyond their (admittedly still valuable) initial guide in whether or not to proceed in the first place. As we have seen, some problems may arise with somatic and/or treatments as well as germline and/or enhancements and so other principles or values seem necessary to break ties or, more strongly, to reveal themselves to be more fundamental moral guides. We hope to have shown how the focus on vulnerability and marginalization can also offer an additional framework for better prioritizing what interventions are permitted or prohibited and under what conditions. Just as Labude et al. (2022) reemphasized the role of the concept of vulnerability in the context of human (germline) genome editing, beyond questions of medical and research ethics, we argue that the concept of vulnerability (and the often related concept of marginalization) can be further useful in narrowing the field of ethical considerations to those that appear most urgent to address – from questions of practical ethics, disability and distributive justice, to potential areas – more pressing and urgent – commanding greater agreement for the purposes of international initiatives (e.g., World Health Organization, 2021c) in promoting public engagement and the basis for global governance.

## Data Availability

The original contributions presented in the study are included in the article/supplementary material, further inquiries can be directed to the corresponding author.

## Footnotes

1 In two senses, the interventions entailed possible enhancement–by design and, perhaps, by accident. The intended change to the CCR5 gene was for resistance to HIV infection which is rare in the human population. Additionally, CCR5-edited mice have shown correlation with enhanced cognitive functioning (Feeney, 2019).

2
https://www.nature.com/articles/d41587-023-00016-6 [accessed 8 December 2023].

3 But, as we highlight, stronger than what would be regarded as Pareto efficiency or Pareto optimality.

4
World Health Organization, 2021c “Human Genome Editing: Recommendations”, p.16.

5 This is particularly the case, as the author notes, with the expansive WHO definition of health as the “state of complete, physical, mental, and social wellbeing” (Kaczmarek, 2019, p.121).

6 In addition, there is an interesting emerging perspective from Lysette Chaproniere (2022) which questions the necessity of an antagonistic ‘disability *versus* enhancement’ relationship.

7 Although one could counter that this is also speculation, that needs to be borne out in due course–and so would share the same provisional nature until this future research would be done.

8
https://www.pharmaceutical-technology.com/news/crispr-gene-therapy-casgevy-secures-approval-in-europe/

9 As it is well-discussed elsewhere, we do not comment on any non-identity issues arising in this statement. For more on this see, see: Ranisch 2020; McMahan, J., & Savulescu, J. 2023.

10 Again, avoiding non-identity considerations, well discussed elsewhere.

11 Indeed, there are many such arguments that the prioritarian approach of incentives and absolute assessments of the worst off, may ignore crucial challenges in terms of adverse effects on an egalitarian ethos and on norms and motivations (Feeney, 2012; Feeney, 2023).

12 Patients were misled and given hope that the gene therapy increased their chances at survival (Williamson, 1982)

13 In 2002, the Human Genome Organisation (HUGO) established that “human genomic databases are global public goods”.

14 In part due to the unique and unrelated impact of the COVID-19 pandemic.

15 The Oviedo Convention was ratified by only 29 states.
